# Roger Guillemin: A Century of Life, A Legacy of Endocrine Discoveries

**DOI:** 10.7759/cureus.68540

**Published:** 2024-09-03

**Authors:** Anshika Baranwal, Prince Rastogi, Abhinav Dixit

**Affiliations:** 1 Physiology, All India Institute of Medical Sciences, Jodhpur, Jodhpur, IND; 2 Endocrinology and Metabolism, All India Institute of Medical Sciences, Jodhpur, Jodhpur, IND

**Keywords:** endocrine gland, pituitary gland, peptide hormone, neuroendocrinology, "historical vignette"

## Abstract

Dr. Roger Guillemin was a French physiologist who won the Nobel Prize in 1977. His pioneering research and fierce competition led to the isolation and identification of hypothalamic hormones, including thyrotropin-releasing factor (TRF), luteinizing hormone-releasing factor (LRF), and somatostatin. These discoveries transformed the knowledge of the role of the hypothalamus in endocrine control. His work not only helped in the advancement of scientific understanding but also directed the field for decades. His research continues to resonate today, shaping modern endocrinology and impacting the global treatment of endocrine disorders.

## Introduction and background

**Figure 1:**
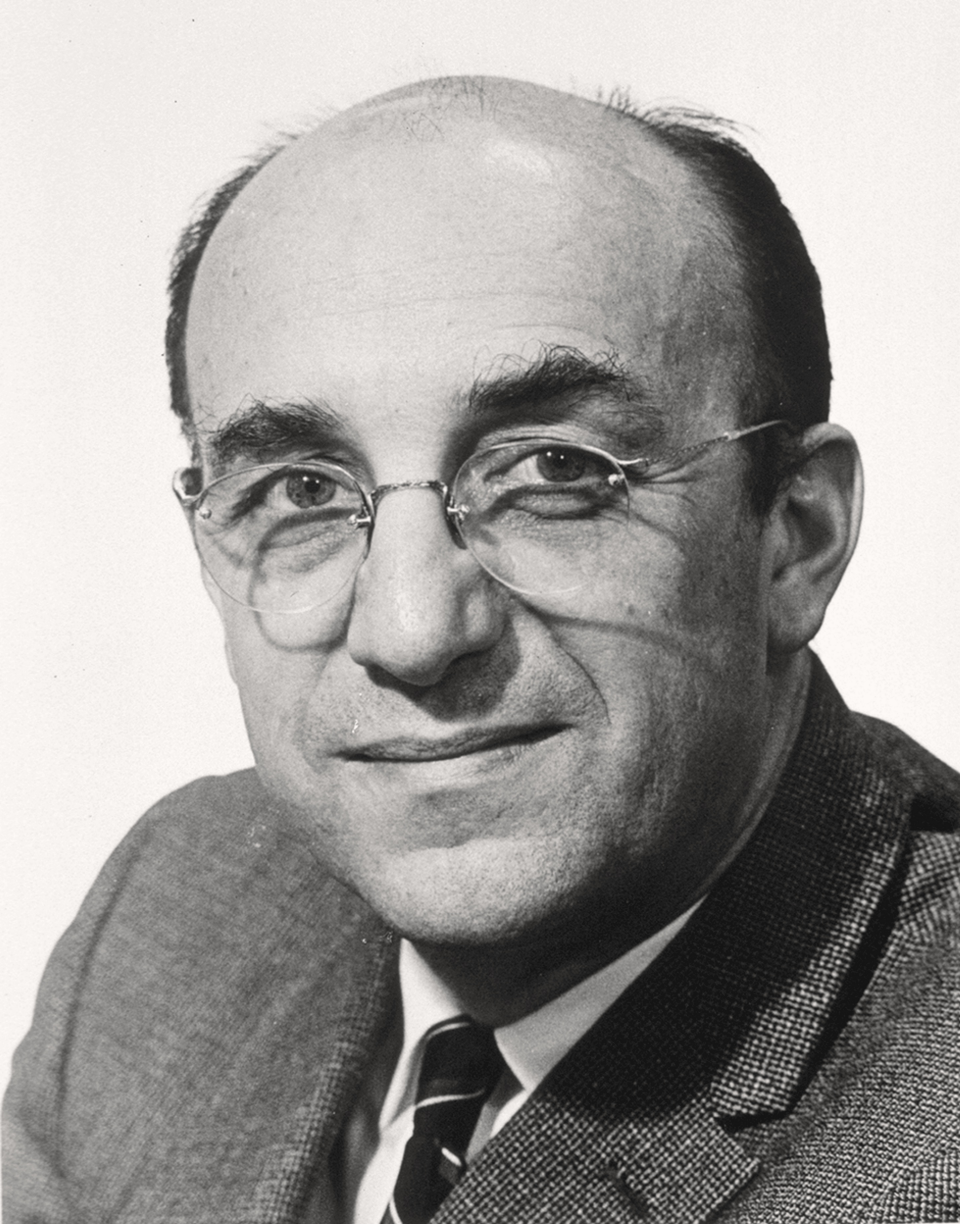
Roger Guillemin Under Creative Commons Public Domain Mark 1.0 license Source: Large Norwegian Encyclopedia [3]. This image is in the public domain.

Roger Guillemin was born on January 11, 1924, in Dijon, France. His journey, which would eventually revolutionize endocrinology, began with his education in Dijon's public schools and lycée. He studied medicine at the Faculté de Médecine of Lyon, earning his M.D. in 1949. Despite the hardships of wartime France, Guillemin developed a strong passion for endocrinology, inspired by renowned clinicians P. Etienne-Martin and J. Charpy, who introduced him to the foundational ideas of this burgeoning field (Figure 1) [1,2].

Roger Guillemin's interest in endocrinology was sparked in 1948 when he attended a Hans Selye lecture in Paris. Roger Guillemin was inspired by Selye's groundbreaking work on the general adaptation syndrome and sought a position in his laboratory. This led to a residency at Selye's Institute of Experimental Medicine and Surgery at the University of Montreal, where he did cutting-edge research on desoxycorticosterone-induced hypertension, culminating in an M.D. dissertation [2].

Roger Guillemin began working in the Department of Physiology at Baylor University College of Medicine in Houston, Texas, in 1953, having completed a PhD program in physiology through a collaboration program between McGill University and the University of Montreal. There for 18 years, Roger Guillemin taught physiology. He made a substantial contribution to the knowledge of the physiological regulation of pituitary gland secretion, especially in reaction to stress, and laid the foundation for later breakthroughs in experimental endocrinology [2].

Roger Guillemin served on several National Institute of Health (NIH) advisory groups for 11 years and was a Council member of the American Endocrine Society from 1969 to 1973. Elected to the National Academy of Sciences in 1974 and the American Academy of Arts and Sciences in 1976, Roger Guillemin has received numerous prestigious awards, including the Gairdner International Award, the Lasker Award in Basic Sciences, and the National Medal of Science. Roger Guillemin has also received honorary degrees from the Universities of Rochester and Chicago, as well as the French government's Légion d'Honneur [2].

He died at the age of 100 in 2024 after making several important discoveries in neuroendocrinology by discovering the chemicals in the brain that regulate hormone synthesis in endocrine glands such as the pituitary and thyroid. His pioneering work unleashed progress in understanding metabolism, reproduction, and development. Guillemin, together with Andrew Schally and Rosalyn Yalow, received the Nobel Prize in Physiology or Medicine in 1977 for his important contributions to peptide hormone studies in the brain [1,4,5].

## Review

Discovery

Guillemin and his colleagues successfully determined the structure of thyrotropin-releasing factor (TRF) in the fall of 1969 after studying millions of sheep brains for more than a decade. This small peptide is generated in the hypothalamus and transferred to the anterior pituitary, where it promotes the production of thyrotropin [6]. Thyrotropin then stimulates the thyroid gland to generate thyroxine, a hormone that controls metabolic activity in almost all bodily tissues. Today, dozens of medications use hypothalamic hormones, including TRF, to treat endocrine disorders and malignancies with a billion-dollar global market value.

The race of discovery spurred by rivalry

Dr. Guillemin made a critical finding in 1954: pituitary cells cultivated in glassware did not produce hormones unless hypothalamus cells were also present. This discovery confirmed the notion of releasing factors, which Dr. Guillemin decided to validate. His research led him to Baylor College of Medicine in Houston, where he went on a difficult mission to extract these hypothesized releasing elements from the hypothalami of animals procured from a kosher slaughterhouse. Despite his efforts, success proved elusive. So, in 1957, Dr. Guillemin joined forces with a promising young researcher, Andrzej V. Schally, also known as Andrew. Together, they pursued the enigmatic releasing factors for five years, but their attempts were met with repeated frustration. Eventually, the collaboration ended, with Dr. Schally relocating to the Veterans Affairs Hospital in New Orleans [6,7].

Undaunted, Dr. Guillemin pursued his goal and recruited two critical team members at Baylor: Dr. Vale, a physiologist, and Roger Burgus, a chemist. These two researchers would form the foundation of his endeavours for the following decade. Dr. Guillemin and Dr. Schally, who were now working separately, realized that they needed substantially more hypothalamus tissue to properly extract the elusive releasing factor. Both researchers developed their laboratory into large-scale processing factories with significant government fund backing. Dr. Guillemin ultimately processed nearly two million sheep hypothalami, whilst Dr. Schally worked with a similar quantity of pig brain tissue [6,7].

The two teams competed fiercely, especially in terms of recognition and credit for scientific breakthroughs. After processing over 270,000 sheep hypothalami, Dr. Guillemin's team was able to isolate a 1-mg sample of TRF, the hormone that regulates the pituitary gland's control over the thyroid. They determined that the TRF molecule was made up of three amino acids: glutamate, histidine, and proline, which Dr. Schally had identified in his pig TRF synthesis in 1966. Schally, who had synthesized all six conceivable tripeptide combinations, discovered that they were physiologically inert, concluding that the hormone's active domain must be located elsewhere in the molecules.

In contrast, Guillemin discovered that these three amino acids made up the complete molecule, together with the remaining contaminants. Despite his team's three-week head start, Guillemin was confronted with a tough decision: publish their discoveries at an upcoming conference or enable other scientists to analyze the structure. Finally, Guillemin chose to reveal the composition, creating a violent rivalry with Schally, who rapidly realized how close his adversary was to discovering the first chemical structure of a brain hormone [6,7].

Schally collaborated with structural scientist Karl Folkers, transferring the tripeptides across state lines to Folkers' lab. Meanwhile, Guillemin contacted Hoffman-La Roche to synthesize the tripeptides that Schally refused to divulge. In 1969, a frantic race to decode TRF's structure ensued, with the conclusion so close that both teams still claimed to be first [7].

Dr. Guillemin's and Dr. Schally's teams competed for seven years before discovering the structure of the TRF. Following several setbacks and successes, both teams discovered the active TRF molecule as pyroGlu-His-Pro-amide. Guillemin's team initially battled with misinterpretations of their infrared data, but Schally's team, despite early mistakes, successfully synthesized the proper structure by August 1969. Although the Schally-Folkers-Bowers team was the first to disclose the proper structure by five weeks, Guillemin's contributions were critical in directing the eventual finding. This result proved the presence of hypothalamus hormones and laid the groundwork for modern endocrinology, securing continuous research funding and developing the discipline [7,8].

Roger Guillemin and Andrew Schally's competition in the search for the brain's hormones lasted more than 20 years. This ongoing struggle began in 1955 when scientists separately proved the connection between the brain and pituitary gland using tissue culture. The race's first milestone was the isolation of TRF in 1969, followed by the discovery of luteinizing hormone-releasing factor (LRF) two years later. The discovery of somatostatin was the third major accomplishment, with Guillemin's team succeeding in 1973 and Schally's in 1976. Last year, their parallel paths culminated in acknowledgement by the Nobel Prize committee in Stockholm [9].

While traditional thinking holds that the TRF milestone was largely a tie, Schally grabbed the lead with LRF, and Guillemin caught up with his work on somatostatin. However, focussing on who "won" this scientific competition ignores the bigger picture. More fascinating are the insights into their combined successes and the enormous influence they had on the area of endocrinology.

The legacy

Dr. Roger Guillemin's legacy in endocrinology is extensive and diverse, involving groundbreaking discoveries, heated scientific competition, and a long-lasting effect on our understanding of hormone control. His research, notably the isolation and identification of hypothalamic hormones like TRF, LRF, and somatostatin, laid the groundwork for current neuroendocrinology.

Pioneering Discoveries

Guillemin's pioneering discoveries on hypothalamic hormones changed our knowledge of hormone control. After repeated disappointments, his team discovered TRF in 1969, providing the first definitive proof of the role of the hypothalamus in directing the pituitary gland, which governs various endocrine activities [6].

Scientific Rivalry and Collaboration

Guillemin and Andrew Schally's intense rivalry led to quick advances in endocrinology. While the rivalry was fierce, especially in the race to decipher the structure of TRF, their race to determine TRF's structure in 1969 demonstrates how scientific rivalry may propel development. Despite their competition, both Guillemin and Schally's contributions were critical, and their work combined pushed the limits of what was known about hypothalamic hormones [6,7].

Impact on Endocrinology

Guillemin's discoveries advanced the understanding of hormone control and opened up new research paths in endocrinology. His contribution secured continuing funding for endocrine research, influencing the field's direction for decades [6-9].

Recognition and Influence

The completion of Guillemin and Schally's parallel journeys in receiving the Nobel Prize demonstrates the long-term impact of their work and their findings, which have a tremendous influence [6].

## Conclusions

His breakthrough contributions to scientific research define Dr. Roger Guillemin's lasting legacy, his crucial role in a remarkable scientific rivalry that fuelled significant progress, and his profound effect on endocrinology. His work has made an everlasting impression on the scientific world, with his discoveries still influencing studies and therapies in hormone-related illnesses.
